# Role of Transbronchial Lung Cryobiopsies in Diffuse Parenchymal Lung Diseases: Interest of a Sequential Approach

**DOI:** 10.1155/2017/6794343

**Published:** 2017-04-20

**Authors:** Benjamin Bondue, Thierry Pieters, Patrick Alexander, Paul De Vuyst, Maria Ruiz Patino, Delphine Hoton, Myriam Remmelink, Dimitri Leduc

**Affiliations:** ^1^Department of Pneumology, Hôpital Erasme, Université Libre de Bruxelles, Brussels, Belgium; ^2^Department of Pneumology, Cliniques Universitaires Saint-Luc, Université Catholique de Louvain, Brussels, Belgium; ^3^Department of Pneumology, AZ Glorieux, Ronse, Belgium; ^4^Department of Thoracic Surgery, Hôpital Erasme, Université Libre de Bruxelles, Brussels, Belgium; ^5^Department of Pathology, Cliniques Universitaires Saint-Luc, Université Catholique de Louvain, Brussels, Belgium; ^6^Department of Pathology, Hôpital Erasme, Université Libre de Bruxelles, Brussels, Belgium

## Abstract

*Background.* Transbronchial lung cryobiopsies (TBLCs) are a promising diagnostic tool in the setting of diffuse parenchymal lung diseases (DPLDs). However, no comparison with surgical lung biopsy (SLB) in the same patient is available.* Methods.* The diagnostic yield and safety data of TBLCs, as well as the result of SLB performed after TBLCs, were analysed in a multicentric Belgian study. A SLB was performed after TBLCs in absence of a definite pathological diagnosis or if a NSIP pattern was observed without related condition identified following multidisciplinary discussion.* Results.* Between April 2015 and November 2016, 30 patients were included. Frequent complications included pneumothorax (20%) and bleeding (severe 7%, moderate 33%, and mild 53%). There was no mortality. The overall diagnostic yield was 80%. A SLB was performed in six patients (three without definite histological pattern and three with an NSIP). The surgical biopsy changed the pathological diagnosis into a UIP pattern in five patients and confirmed a NSIP pattern in one patient.* Conclusion.* TBLCs are useful in the diagnostic work-up of DPLDs avoiding a SLB in 80% of the patients. However, surgical biopsies, performed as a second step after TBLCs because of an indefinite diagnosis or a NSIP pattern, provide additional information supporting the interest of a sequential approach in these patients.

## 1. Introduction

Diffuse parenchymal lung diseases (DPLDs) are a heterogeneous group of diseases with a variable amount of fibrosis and inflammation. For prognostic and therapeutic purposes, a precise diagnosis is required. This is performed by the confrontation of clinical, radiological, and, when available, pathological data during a multidisciplinary discussion (MDD) [1, 2]. If the high resolution CT (HRCT) scan is typical for a usual interstitial pneumonia (UIP), no biopsy of any kind is indicated to confirm a UIP pattern [2]. In the other cases, if a lung biopsy is required, a surgical lung biopsy (SLB) is recommended as conventional transbronchial biopsy using forceps provides generally too small specimens to bring information in the diagnosis of the majority of DPLDs [1, 2].

However, SLB is an invasive procedure with significant comorbidities requiring hospitalization for a few days and a systematic chest drainage. The rate of postoperative mortality of SLB ranges between 0% and 3,6% [3–7]. Therefore, the indication of SLBs has to be carefully taken by the multidisciplinary team and the development of less invasive technique has emerged: thoracic surgeons, who originally performed an open lung biopsy, developed the VATS surgery, and, for a few years, transbronchial lung cryobiopsy (TBLC) has been described for the diagnosis of DPLDs.

Compared to conventional transbronchial biopsies, TBLCs provide specimens significantly larger with approximately three to six times the amount of alveolar tissue without crush artifacts [8–10]. As a consequence, the diagnostic usefulness (and the possibility of identifying a UIP pattern) of transbronchial cryobiopsy clearly exceeds that of conventional transbronchial biopsy obtained with forceps [9–14]. In a recent meta-analysis including 994 patients from 15 studies, Ravaglia and colleagues revealed an overall diagnostic yield of 81% for TBLCs in DPLDs [6]. Bronchial bleeding and pneumothorax are the main complications after TBLCs. The probability of developing a pneumothorax was 6% in the meta-analysis of Ravaglia et al. [6] but reached up to 28% in some studies depending of the distance between the chest wall and the cryoprobes, the underlying disease, and the experience of the endoscopist [14]. Severe bleeding is rare and clinically significant bleeding (severe and moderate) can be safely controlled by the use of a Fogarty balloon [6, 14].

A remaining question is how to position TBLCs compared to SLB in the assessment of an undefined DPLD. However, no data compared directly, in the same patient, the diagnostic yield of SLB and TBLC. As performing these two techniques in a patient at the same time point could present some ethical limitations, we rather evaluated the yield of a sequential approach in which a SLB is performed after TBLCs when this latter technique is inconclusive or provides an unspecific diagnosis (including a histological pattern of nonspecific interstitial pneumonia NSIP).

## 2. Material and Methods

### 2.1. Subjects

A multicentric prospective observational study was performed between April 2015 and November 2016 in three Belgian hospitals and was approved by the ethical committees of the nonleading hospitals and by the leading ethical committee of the Erasme hospital (ref P2015/192). Written informed consent for participation in the study was obtained from each patient before any study procedure. Patients were included if they present a DPLD requiring a lung biopsy as evaluated by the multidisciplinary team. All indications and biopsy results were discussed within multidisciplinary teams experienced in management of interstitial lung diseases. These multidisciplinary teams include at least one chest physician, one pathologist, one thoracic radiologist, one specialist in internal medicine, or one rheumatologist. Patients had to be at least 18 years old, with a forced vital capacity (FVC) higher than 50% of predicted value, a diffusing capacity for carbon monoxide (DLCO) higher than 30% of predicted value, and a pulmonary systolic arterial pressure estimated by echocardiography less than 40 mmHg or measured by a right heart catheterization showing a mean pulmonary artery pressure (mPAP) less than 25 mmHg at rest. Exclusion criteria included also coagulopathy (platelet count < 100000/mm^3^, prothrombin time international normalized ratio-INR > 1.5, and activated partial thromboplastin time-APTT > 35), hypoxemia (PaO_2_ < 55 mmHg on room air), or hypercapnia (PaCO_2_ > 45 mmHg), severe underlying cardiac disease, and suspicion of pleuroparenchymal fibroelastosis based on the HRCT aspect (presence of a dense pleural and subpleural consolidation with a reticular pattern, predominantly in the upper lobes) [15]. Collagen vascular disease-associated interstitial lung disease (CVD-ILD) and drug-induced interstitial lung disease (D-ILD) were not formally excluded but we try to avoid lung biopsies in these patients as the diagnosis can be achieved by other means.

### 2.2. Experimental Protocol

Patients could choose between SLBs and TBLCs taking into account the advantages and disadvantages of both techniques. If TBLCs were preferred, patients were included in the study and informed of the possibility of having a surgical biopsy following the endoscopic procedure in case of unclear pathological diagnosis or a histopathologic pattern suggestive of NSIP. Indeed, idiopathic NSIP, which is nowadays considered as a specific entity [1], is defined histologically by varying amounts of interstitial inflammation and fibrosis with a uniform appearance [16]. However, the assessment of this pathological uniformity is based on the analysis of SLBs that are bigger than TBLCs and performed in different lobes. Therefore, TBLCs could miss other specific features such as UIP lesions that could change the final diagnosis and the prognosis of the patient [17].

**Figure 1:**
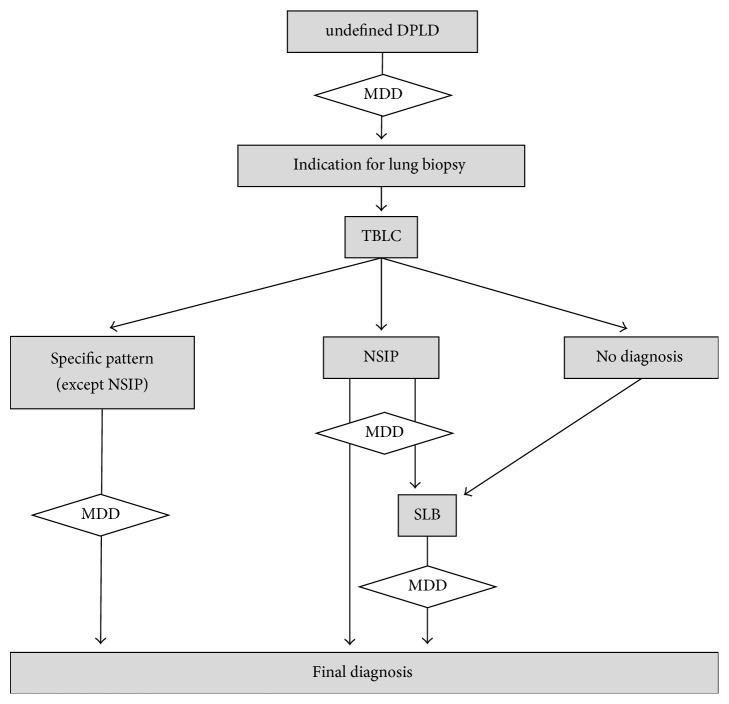
Algorithm summarizing the study protocol. DPLD: diffuse parenchymal lung disease; MDD: multidisciplinary discussion; SLB: surgical lung biopsy; NSIP: nonspecific interstitial pneumonia; TBLC: transbronchial lung cryobiopsy.

Of note, a SLB was not performed in patients with a histological NSIP pattern following TBLCs if a related condition (such as a hypersensitivity pneumonitis) was identified by the multidisciplinary team according to the analysis of all the available data. This includes the HRCT pattern, cellularity of the bronchoalveolar lavage, specific IgG (precipitins), autoantibodies, drugs, and environmental and occupational exposures. SLBs were obtained by video-assisted thoracoscopic surgery (VATS) and performed in at least two different lobes as recommended in the guidelines for patients with suspected idiopathic pulmonary fibrosis (IPF) [2]. The design of the experimental protocol is summarized in Figure 1.

### 2.3. Bronchoscopy and Cryobiopsy

Procedures were performed under general anesthesia, intubation with a rigid bronchoscope, and jet ventilation in a bronchoscopy suite with standard monitoring and sedation procedure. Antiaggregant and anticoagulant therapies were stopped before the procedure (seven days for acetylsalicylic acid, dabigatran, rivaroxaban and apixaban, ten days for clopidogrel, six hours for unfractionated heparin, and 24 hours for low molecular weight heparin). Erbe manufactures two sizes of flexible cryoprobes (1,9, and 2,4 mm in diameter). For TBLCs, participating hospitals used the cryoprobe of 2.4 mm diameter (ERBE, Germany). The cryoprobe operates using the Joule-Thomson effect, in which compressed gas (CO_2_) undergoes an adiabatic expansion and rapidly cools the probe tip to −45°C within several seconds. The biopsies were obtained by insertion of the cryoprobe through the working channel of a flexible bronchoscope placed into the rigid bronchoscope. Biopsies were performed in the most affected areas except where honeycomb changes predominated. We attempted to obtain four biopsies from two different segments of the same lobe. For each biopsy, the cryoprobe was pushed under fluoroscopic guidance to the distal parenchyma and the probe was withdrawn one-two cm from the thoracic wall. Once in position, the probe was cooled for five to six seconds; then the probe and the bronchoscope were removed en bloc out of the airway, and the frozen specimen was thawed first in saline at room temperature and afterwards transferred to formalin for fixation. To control potential severe bleeding, a noninflated Fogarty balloon, previously placed in the lobar bronchus close to the sampled segment, was inflated immediately after biopsy and then deflated in case of absence of hemorrhage. The bleeding was scored as follows: score 0 if no bleeding, score 1 if bleeding stopped with aspiration only and/or insufflation of the Fogarty balloon less than 5 minutes, score 2 if bleeding stopped by cold saline instillation and/or prolonged use of the Fogarty balloon (more than 5 minutes), and score 3 if life-threatening bleeding requiring any of the following: embolization, selective bronchial intubation, transfusion, admission in an intensive care unit (ICU), or resulting in death or prolonged hospitalization. Within 3 h after the procedure, a chest X-ray was obtained to exclude pneumothorax. All patients stay in the hospital for one night after the procedure for monitoring (the aim of this monitoring was to identify relapse of bleeding and subacute pneumothoraces).

### 2.4. Biopsy Specimens

Biopsy specimens were fixed in 10% formalin and embedded in paraffin. Hematoxylin and eosin as well as Masson's Trichrome, Giemsa, staining was performed and immunostaining against pancytokeratins was performed. Specimens were reviewed by a pathologist expert in interstitial lung disease. If case of doubt regarding the diagnosis, two other pathologists experts in interstitial lung diseases could review the specimens to obtain a consensual diagnosis.

## 3. Results

30 patients were included in the study. Patient's characteristics are summarized in Table 1. The chest HRCT showed an inconsistent UIP pattern in the majority of the patients (80%) whereas a typical UIP pattern was present in one patient. In this patient, pleural plaques were also noticed and the lung biopsy was performed to count asbestos bodies (differential diagnosis between idiopathic pulmonary fibrosis and asbestosis). Of note, cryobiopsies were sufficient for mineralogical analysis. 333 asbestos bodies per gram of dry tissue were identified and the diagnosis of asbestosis was therefore rejected (for an asbestosis, 5000 asbestos bodies per gram of dry tissue or more are required) [18].

The mean number of biopsies by patient was 4,2 (range 2–5) and the mean specimen area was 17 mm^2^ (range 10–40 mm^2^). Cryobiopsies were mainly performed in the lower lobes (23 patients) whereas the upper lobes were biopsied in six and the middle lobe in one patient. Main complications were pneumothorax and bleeding (Table 2). Pneumothorax occurred in six patients (20%) and required chest tube in three patients (50%), simple aspiration in two patients (33%), and only observation in one. Bleeding was in the majority of the cases mild (53%, grade 1) and moderate (33%, grade 2). Severe bleeding occurred in two patients and required a prolonged use of the Fogarty balloon and injection of cold saline. One of these patients was also admitted in ICU for overnight surveillance. With respect to the limited number of patient, we cannot definitely conclude but there was no clear relationship between the number or the size of the biopsies and the risk of pneumothorax and bleeding (supplemental data, Figure S1 in Supplementary Material available online at https://doi.org/10.1155/2017/6794343).

Interestingly, the mean hospitalization time after the procedure was 1,3 days taking into account the mandatory overnight monitoring. No significant chest pain among patients without pneumothorax was reported as well as prolonged air leak, infection, acute exacerbation, or death.

**Figure 2:**
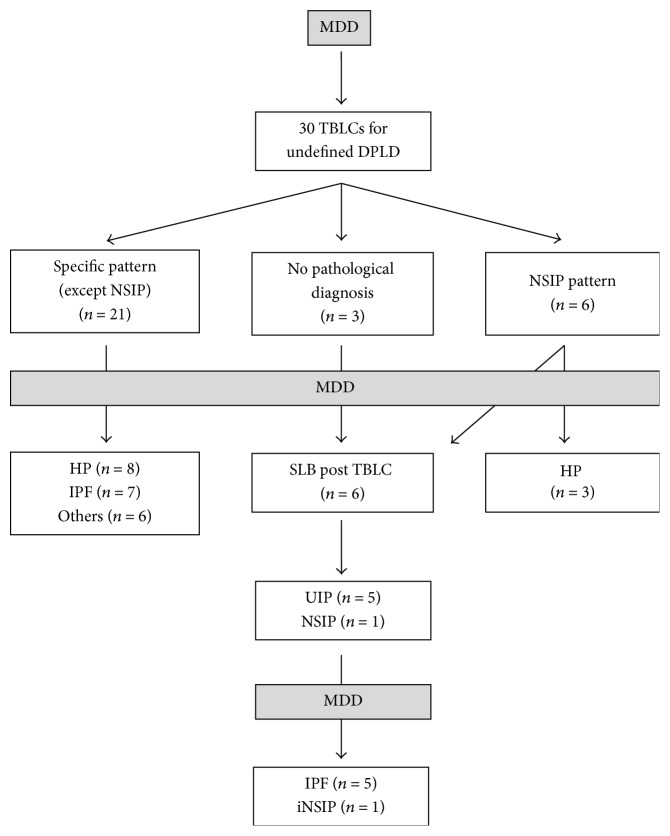
Detailed diagnoses obtained after TBLCs (and SLBs when applicable).

The overall diagnostic yield of TBLCs was 80% (24/30). This corresponds to patients with either a specific histological pattern other than an NSIP or patients with an NSIP for which this pattern could be related to a specific condition. The detailed diagnoses obtained are summarized in Figure 2 and Table 2. A specific histological pattern (other than NSIP) was identified in 21 patients. Among these, the final diagnosis, after discussion within the multidisciplinary team, was IPF in seven patients and hypersensitivity pneumonitis (HP) in eight patients (the other less frequent diagnoses are listed in Figure 2). In three patients, no definite histological diagnosis was identified, and, in six patients, an NSIP pattern was observed (four cellular and two fibrotic NSIP patterns). In three out of the six patients with an NSIP pattern, the presence of this histological pattern helped the multidisciplinary team to identify a hypersensitivity pneumonitis taking into account the other available clinical data such as exposures, BAL lymphocytosis, presence of specific antibodies (precipitins), and radiological features. Therefore, in these patients, no surgical biopsy was proposed after TBLCs.

**Figure 3:**
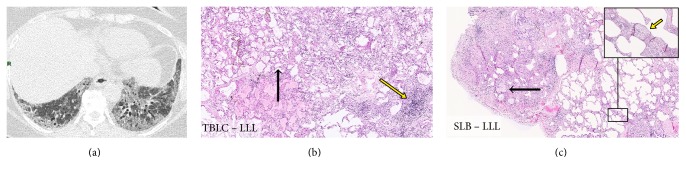
Illustration of the additional information obtained from surgical biopsy after cryobiopsies showing an NSIP pattern. A 75-year-old woman with idiopathic interstitial pneumonia had a chest CT scan showing an inconsistent UIP pattern (subpleural and basal reticular opacities, traction bronchiectasis, and ground glass opacities with an extent greater than reticular abnormalities without honeycombing) (a). TBLCs were performed in the left lower lobe and the pathological analysis identified an NSIP pattern (hematoxylin and eosin staining, magnification ×40) (b). The NSIP pattern was characterized by the presence of an interstitial septal thickening preserving the alveolar architecture, black arrow, with chronic inflammatory infiltrate, red arrow. Neither honeycombing nor fibroblastic foci were observed. A surgical lung biopsy was then performed in the same patient, in the same lobe (left lower lobe) showing a UIP pattern (hematoxylin and eosin staining, magnification ×40) (c). The photomicrography shows the presence of fibrotic changes with honeycombing (black arrow), heterogeneous architectural destructions and fibroblastic foci (enlargement and red arrow, magnification ×200). Those features were not observed on the cryobiopsy.

On the contrary, a surgical lung biopsy was performed in the three other patients with an NSIP pattern as well as in the three patients without a clear histological diagnosis. Among these six patients, the histological assessment of the surgical biopsy confirmed a NSIP pattern in only one patient and showed a UIP pattern in the remaining five patients. Of note, the patient with a surgically confirmed NSIP pattern had also a NSIP pattern in lung tissue obtained by cryobiopsy. Interestingly, the additional information provided by surgical biopsies came from specimens obtained from the same lobes as those selected for cryobiopsies (as illustrated in Figure 3). After discussion within the multidisciplinary team, the final diagnosis for these six patients was IPF in five and an idiopathic NSIP in one patient.

## 4. Discussion

Our study confirms the usefulness of TBLCs in the multidisciplinary diagnosis of DPLDs with an overall diagnostic yield of 80%. Thus a surgical lung biopsy could be avoided in 80% of the patients who benefit from cryobiopsies. This result is in agreement with previously published data and recent meta-analyses showing a global diagnostic yield of cryobiopsies of around 80% [6, 12–14, 19–22]. The most frequent diagnoses obtained were IPF and HP. No CVD-ILD and D-ILD were identified in our study. This is explained by the small size of our cohort and because we avoid doing lung biopsies in patients with these suspected diseases. Indeed, lung biopsies will show different patterns (including UIP and NSIP patterns), and the diagnosis can generally be achieved on the basis of other clinical data (clinical history, presence of extrapulmonary signs/symptoms, presence of autoantibodies, and results of extrapulmonary investigations). Another concern could be the capacity of cryobiopsies to identify overlap of histologic patterns and lymphoid nodules often proved to be related to CVD-ILD [1].

Compared to SLB, other benefits of TBLCs are lower morbidity and shorter hospitalization time (slightly more than one day after the procedure in our study) supporting the recently reported cost-effectiveness of this technique [20]. We also confirm that bleeding and pneumothorax are the main complications following TBLCs. Bleeding is mostly mild to moderate. Severe bleeding can occur and is controlled by the use of a Fogarty balloon. Pneumothorax rate is around 20%, as expected when biopsies are performed between 1 and 2 cm of the thoracic wall, but the placement of a chest tube is not always mandatory [6, 14, 23]. There was no clear relationship between the number, the size, or the localisation of the biopsies and the risk of pneumothorax and bleeding. However, the study was not designed to analyse those relationships. Indeed, the cohort was too small with only one patient having two biopsies whereas the others had four or five biopsies, mostly in the lower lobes. This resulted in a significant underrepresentation of many subgroups to allow accurate comparisons and formal conclusions.

In order to definitely compare the ability of TBLCs to identify accurately a specific histological pattern, both techniques (TBLCs and SLBs) should be performed in the same patients. However, such data have not been published so far and raise ethical limitations. Of interest, our study provides data from some patients having a sequential approach with both procedures (TBLCs first, and afterwards SLB) evaluating the added value of SLB to TBLC alone in terms of diagnostic yield. As we thought it is unethical to perform the two procedures in all patients, we selected two specific situations in which we hypothesized that SLB could provide complementary information, that is, when the histological pattern was inconclusive or showed a NSIP (except if a related condition/etiology could be identified supporting the accuracy of this histological diagnosis). The rationale for performing SLB after TBLCs in such situations is based on retrospective analysis showing a slightly lower diagnostic yield for TBLC than for SLB (82.8% versus 98.7%, resp.) [6]. Moreover, in 26% of IPF patients who benefit from a SLB, a discordant UIP pattern is present with a UIP pattern in a lobe and a NSIP in another one [17]. The accuracy of TBLCs to identify such discordant patterns has not been studied so far and could be lower than for SLB taking into account the notion that the sizes of the specimens are lower and generally performed in only one lobe. Therefore, in the present study, we proposed to perform a SLB after TBLC in patients with an unclear histological pattern following TBLCs or when a NSIP pattern was present without related condition.

This situation occurred in six patients out of 30 (20%). Interestingly, in five out of these six patients (83%) the SLB provided additional information. Indeed, the histological diagnosis was a typical UIP pattern, changing significantly the diagnosis, the prognosis, and the treatment of these five patients, as the final multidisciplinary diagnosis was IPF. In the majority of these five patients, the cryobiopsies were performed in the lower lobes (4/5) whereas the surgical biopsy was performed in two lobes (the upper and the lower lobes). Interestingly, it is not the surgical specimen obtained from the noncryobiopsied lobe who induces the change in the diagnosis (as illustrated in the Figure 3). In fact, SLB provides more information mostly because the size of the biopsies was greater and therefore more pathological features could be identified. Finally, idiopathic NSIP was only diagnosed in one patient out of 30 (3%) confirming that this diagnosis is relatively rare.

In conclusion, our data confirm the role of TBLCs for the multidisciplinary diagnosis of DPLDs when a lung biopsy is required. Morbidity and hospitalization time are lower than after surgical lung biopsy [6]. The diagnostic yield is around 80%, thus avoiding a SLB in 80% of the cases. Therefore, our data support that TBLCs should be the first line procedure when the analysis of a lung biopsy is required. However, when an inconclusive result or a NSIP pattern without related condition (idiopathic NSIP) is obtained, a SLB performed after TBLC could provide complementary information. Altogether, those data, even preliminary and limited, support the concept of the sequential approach as made for the mediastinal staging of lung cancer. In this approach, endoscopic procedure (TBLCs) is performed first and SLB is reserved for inconclusive or NSIP results after cryobiopsies. Other studies are required to confirm these preliminary data and to support this sequential approach. In the meanwhile, chest physicians should cautiously interpret NSIP results, especially in idiopathic condition.

## Supplementary Material

For each patient, the number of cryobiopsies, the bleeding score, and the presence of a pneumothorax were recorded. The total area of the biopsies was also measured by the addition of the surface of each biopsy. These data were analyzed using the Prism6 software (GraphPad Software). The Kruskal-Wallis test was used for multiple comparisons between groups and a *P* value of less than 0.05 was considered significant.

## Figures and Tables

**Table 1:** Clinical characteristics of the patients (*N* = 30).

Gender	Male *N* (%)	14 (47)

Age, years	Median (range)	62 (26–80)

Smoking history	Current *N* (%)	3 (10)
	Former *N* (%)	17 (57)
	Never *N* (%)	10 (33)

BMI	Median (range)	29 (17–39)

FVC, % predicted value	Median (range)	73 (54–104)

DLCo, % predicted value	Median (range)	50 (30–76)

HRCT	Typical UIP *N* (%)	1 (3)
	Possible UIP *N* (%)	5 (17)
	Inconsistent UIP *N* (%)	24 (80)

Number of biopsies/patient	Mean (range)	4,2 (2–5)

Size of biopsies (mm^2^)	By specimen Mean (range)	16,6 (9,5–40)
	By patient Mean (range)	71,0 (42–106)

BMI: body mass index; HRCT: high resolution chest tomography; FVC: forced vital capacity; DLCO: diffusion capacity of the lung for carbon monoxide.

**Table 2:** Complications and histological diagnosis of the TBLCs (*N* = 30).

Hemorrhage *N* (%)	Grade 0	2 (7)
	Grade 1	16 (53)
	Grade 2	10 (33)
	Grade 3	2 (7)

Pneumothorax	All	6 (20)
*N* (% total patients)	Requiring chest drainage	3 (10)

Detailed histological diagnosis *N* (%)	HP	8 (27)
	UIP	7 (23)
	NSIP	6 (20)
	Sarcoidosis	2 (7)
	DIP	1 (3)
	Amyloidosis	1 (3)
	Eosinophilic pn.	1 (3)
	Adenocarcinoma	1 (3)
	Undetermined	3 (10)

HP: hypersensitivity pneumonia; UIP: usual interstitial pneumonia; NSIP: nonspecific interstitial pneumonia; DIP: desquamative interstitial pneumonia; eosinophilic pn.: eosinophilic pneumonia.

## Abbreviations

APTT: Activated partial thromboplastin time
BMI: Body mass index
CVD-ILD: Collagen vascular disease-associated interstitial lung disease
D-ILD: Drug-induced interstitial lung disease
DLCO: Diffusion capacity for carbon monoxide
DPLD: Diffuse parenchymal lung disease
FVC: Forced vital capacity
HP: Hypersensitivity pneumonitis
HRCT: High resolution computed tomography
ICU: Intensive care unit
INR: Prothrombin time international normalized ratio
IPF: Idiopathic pulmonary fibrosis
MDD: Multidisciplinary discussion
mPAP: Mean pulmonary artery pressure
NSIP: Nonspecific interstitial pneumonia
SLB: Surgical lung biopsy
TBLC: Transbronchial lung cryobiopsy
UIP: Usual interstitial pneumonia
VATS: Video-assisted thoracoscopic surgery.
