# Supplementary material for: Role of Transbronchial Lung Cryobiopsies in Diffuse Parenchymal Lung Diseases: Interest of a Sequential Approach
Source: Pulm Med. 2017 Apr 20;2017:6794343. doi: 10.1155/2017/6794343 (PMC5415669; doi:10.1155/2017/6794343)
Supplement: Supplementary file 1 — For each patient, the number of cryobiopsies, the bleeding score, and the presence of a pneumothorax were recorded. The total area of the biopsies was also measured by the addition of the surface of each biopsy. These data were analyzed using the Prism6 software (GraphPad Software). The Kruskal-Wallis test was used for multiple comparisons between groups and a P value of less than 0.05 was considered significant. [file 6794343.f1.docx]

**Supplemental data**

Figure S1

**Figure S1** Analysis of the relationship between the number (A) and the size (B) of the biopsies (evaluated by the total area of the biopsies obtained in a patient) and the risk of pneumothorax. No significant differences were observed between the group with and without pneumothorax. Analysis of the relationship between the number (C) and the size (D) of the biopsies and the risk of bleeding. No significant differences were observed (only a trend to higher bleeding score in patients with the highest biopsies area).
